# Digitally Fabricated Fixed Restoration for Immediate Replacement After Extraction and Splinting the Adjacent Mobile Teeth

**DOI:** 10.1155/2024/9962990

**Published:** 2024-10-21

**Authors:** Jian Li, Shenghao Xue, Ke Li, Tianyi Huyan, Ting Jiang

**Affiliations:** ^1^Department of Prosthodontics, Peking University School and Hospital of Stomatology & National Center of Stomatology & National Clinical Research Center for Oral Diseases & National Engineering Research Center of Oral Biomaterials and Digital Medical Devices, Beijing, China; ^2^Department of Dental Laboratory Center, Peking University School and Hospital of Stomatology & National Center of Stomatology & National Clinical Research Center for Oral Diseases & National Engineering Research Center of Oral Biomaterials and Digital Medical Devices, Beijing, China

**Keywords:** CAD-CAM technique, partial edentulous, periodontitis, prosthodontics, resin-bonded fixed partial denture, splint

## Abstract

**Objective:** This study is aimed at detailing a technique for digitally fabricated fixed restoration for both immediate replacement after extraction and splinting of the adjacent mobile teeth.

**Clinical Considerations:** Demand for a fixed restoration of the missing teeth in the mandibular anterior region is very common but sometimes problematic for dentists. It often happens that there is significant loosening of the remaining teeth adjacent to the missing tooth, which should be splinted. This case report describes a digitally fabricated fixed restoration for both replacing lost teeth and splinting periodontally compromised mobile teeth. With the CAD-CAM technique, patients can receive the restoration immediately after the extraction. The digital design and fabrication of the restoration were elaborated using the restorative process of a patient with a mandibular anterior tooth to be extracted and multiple loose lower anterior teeth. The immediate restoration process and the pontic modification when the extraction socket had healed were also described.

**Conclusions:** The digital process avoids the nudging of the loose tooth by the traditional impression technique, improves the accuracy of the restoration, and avoids undue stress on the loose tooth when the restoration is in place. In addition, this method allows the patient to obtain a fixed restoration immediately after extraction, and if damage occurs after the restoration has been cemented, such as fracture or debonding, a new restoration can be prepared before the patient's return visit.

**Clinical Significance:** This minimally invasive restorative method achieved the fixation of loose teeth while restoring the missing teeth, but the success of the treatment needed long-time observation.

## 1. Introduction

Periodontitis is the leading cause of tooth loss in adults [1, 2]. Usually, when tooth loss is due to severe periodontitis, the periodontal support tissue of the adjacent teeth is compromised [3]. Demand for a fixed restoration of the missing teeth in the mandibular anterior region is very common but sometimes problematic for dentists. Teeth loss accompanied with alveolar bone defect, as well as the compromised periodontal conditions of adjacent teeth, presents a significant clinical problem, which is hard to be rehabilitated with either implants [4] or fixed partial dentures (FPDs) [5]. In these situations, the main treatment strategy involves not only the replacement of the lost teeth to restore oral function and esthetic appearance but also the remedy of the deteriorated periodontal conditions of the adjacent remaining teeth. In addition, the convenience of maintenance and management after restoration is indispensable.

The investigations on periodontally compromised splinted teeth showed good long-term prognosis when patients adhered to supportive periodontal therapy [6–8]. Moreover, satisfactory results can be found in the clinical application of glass fiber–reinforced composite splints to stabilize mobile teeth, which has been proven useful in the improvement of the periodontal condition [9]. The application of glass fiber–reinforced composite-based resin-bonded fixed partial dentures (GFRC-RBFPDs) to replace a single lost anterior tooth was also satisfying [10, 11], which had an acceptable survival rate of 85.6%. In 2016, a 4-year clinical observation evaluated the outcome of using GFRC-RBFPDs to replace lost teeth while simultaneously stabilizing adjacent mobile teeth [12]. In the fourth year, the complete survival rate was 89.7%, and the functional survival rate was 92.3%. The periodontal condition of the adjacent teeth was shown to have been improved. In addition, the failure was found mainly from the fracture of the connector of GFRC-RBFPDs. Looking into the reasons, the conventional impression might nudge or tilt the mobile teeth which influenced the adaptation of GFRC-RBFPDs and induced harmful stress on the teeth when the restoration was in place or being bonded. The fabrication technique of stacking also limited the fit between GFRC-RBFPD and the preparation. The stiffness of the restoration was also affected.

The introduction of computer-aided design and computer-aided manufacturing (CAD-CAM) techniques has allowed prosthodontists to take the advantage of intraoral digital scans which will improve the precision of the impression taken on loose teeth [13]. However, so far, the reports describing a digitally fabricated fixed restoration for both replacing lost teeth and splinting periodontally compromised mobile teeth are absent. Now, in a clinical research project supported by the Program for New Clinical Techniques and Therapies of the Peking University School of Stomatology (No. PKUSSNCT-21A08), the long-term success rate of this type of digitally fabricated fixed restoration and the improvement of the periodontal condition of fixed teeth are investigated. Ethical approval was obtained from the biomedical ethics committee of Stomatology Hospital, Peking University (ethical batch number: PKUSSIRB-202171199).

## 2. Technique

**Figure 1:**
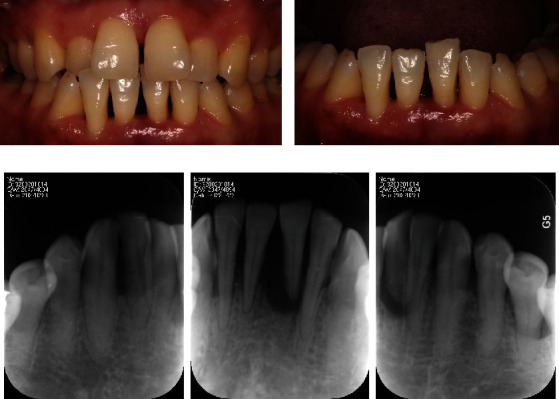
Intraoral photograph before the extraction of the mandibular left central incisor. (a) Labial view of anterior teeth. (b) Protrusion of the mandibular left central incisor. (c) Periapical film of the mandibular anterior teeth.

A 38-year-old female patient who had to replace the mandibular left central incisor with a mobility degree of III was enrolled once she provided written informed consent (Figure 1). She had just received nonsurgical periodontal treatment. The scaling and root planning controlled the periodontal inflammation effectively. Then, she followed the advice of her doctor in the periodontology department and came to the prosthodontic department to ask for a denture to replace the mandibular left central incisor. In the mandible, the right central incisor and bilateral lateral incisors also had a mobility degree of I or II. The periapical film showed that the bone on the mesial side of the mandibular right central incisor or left lateral incisor and on the distal side of the mandibular right lateral incisor reduced to the apical third of the root. And the bone on the distal side of the mandibular right central incisor and on the mesial side of the right canine was absorbed to the cervical third of the root (Figure 1(c)). The patient believed that it was unacceptable to wait for a period of time to repair the missing teeth, whether it was due to the need to make a denture or waiting for the restoration of extraction socket. Therefore, a digitally fabricated fixed restoration for both immediate replacement after the extraction and splinting of the adjacent mobile teeth became her suitable rehabilitation treatment. The process of restoration treatment is shown in Table 1.

### 2.1. Digital Design and Fabrication of FPD

**Figure 2:**
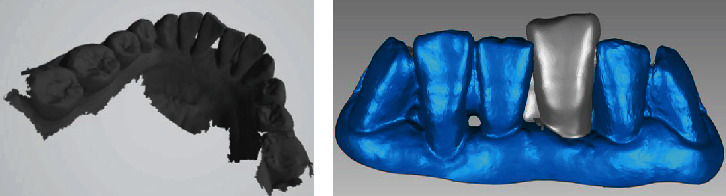
Digital impression and digital model. (a) Intraoral digital scan of the mandible. (b) Three-dimensional digital model.

**Figure 3:**
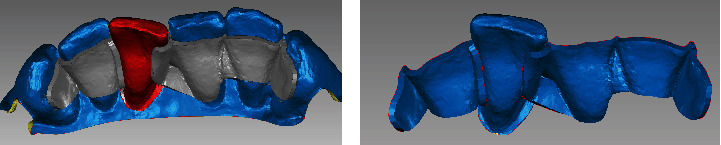
Design the denture with the reverse engineering software Geomagic Studio 2013. (a) Design the denture on the digital model. (b) Three-dimensional design of the denture.

Using an intraoral scanner (3Shape Trios, 3Shape A/S, Copenhagen, Denmark), intraoral digital scans of the maxilla, mandible, and maximal intercuspal position before the tooth extraction were made. Save the digital scans as a 3Shape exchange format (stereolithography (STL)) file (Figure 2). With the reverse engineering software Geomagic Studio 2017 (Geomagic Inc, North Carolina, United States), a three-dimensional digital model was obtained from the STL file of the digital impression, on which the FPD was designed (Figure 3). The design needs to comply with the requirements of fixed periodontal splints, which anchor loose teeth to firm healthy teeth to form a new masticatory unit that allows the abutment teeth to fully exploit their periodontal tissue potential to compensate for the mobile teeth with periodontally compromised conditions, reducing the occlusal force burden on the affected teeth and allowing them to rest physiologically for tissue repair and healing. In this case, the remaining three mandibular incisors and bilateral lower canines need to be splinted together. The wing-plates were placed on the lingual side of the teeth being fixed and the abutment teeth. Since the undercut on the proximal surfaces adjacent to the missing tooth might hinder the seating of the pontic, the undercut that was near the neck of the mandibular right central incisor and left lateral incisor was filled in the direction of the insertion path. For this patient, the shape, and the position of the pontic fully mimicked the loose teeth to be removed. To design the plates of the denture, the border lines were drawn on the lingual surfaces of the anterior teeth. In order to increase the bonding area, the range of the wing-plates should be expanded as much as possible. The upper line should not extend beyond the line angle of incisal surface and lingual surface, usually at least 1 mm below the incisal border of the anterior teeth. The lower line was at least 1 mm above the gingival margin. All the margins and the shape of the plate were tailored and designed to get uniform contact with the lingual and proximal surfaces of the mandibular anterior teeth. The thickness of the plate was 1.0 mm. The final design of the prosthesis was saved in an STL format file.

**Figure 4:**
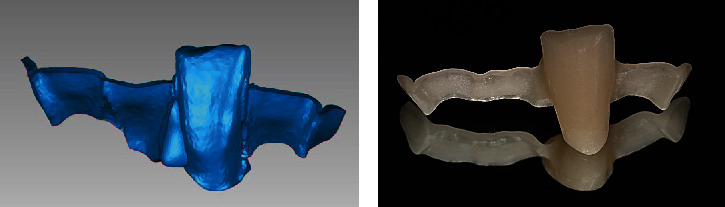
RBFPD was milled by the CAM machine. (a) Labial view of the design of the denture. (b) The fabricated denture.

**Figure 5:**
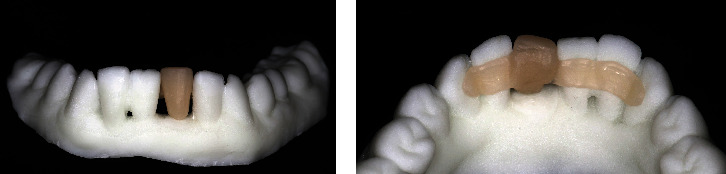
RBFPD was tried on the printed model. (a) Labial view of the denture seated on the printed model. (b) Lingual view of the denture seated on the printed model.

Import the STL file into the milling software in the dental laboratory. The milling resin with an elasticity modulus of 140 MPa (polymethylmethacrylate block, Huge Dental Material Corporation, Shandong, China) was used to fabricate the FPD with the CAM machine (Organical Multi S & Changer 20, Organical CAD/CAM GmbH, Berlin, Germany) . The milled FPD was checked for integrity, surface roughness, and edge moving (Figure 4). Then, it was tried on the printed model (Figure 5). Only with a little amount of correction in the proximal surfaces of the pontic, the FPD was fully in place and the marginal adaptation was satisfactory.

### 2.2. Immediate Cementation of FPD After the Extraction

**Figure 6:**
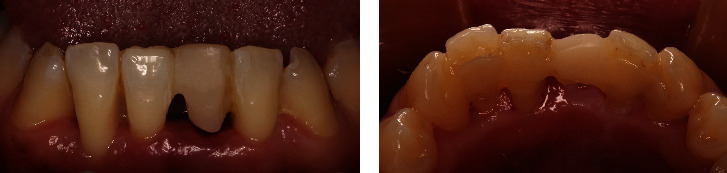
Cement the denture right after the mandibular left central incisor was extracted and the bleeding in the socket stopped. (a) Labial view of the cemented denture. (b) Lingual view of the cemented denture.

After the extraction of the mandibular left central incisor and the cessation of bleeding from the extraction socket, the restoration was tried on until fully seated under a rubber barrier (Figure 6). Then, the denture was polished outside the mouth. The lingual surfaces and proximal surfaces of the mandibular anterior teeth within the scope of the denture contacting were etched with 35% orthophosphoric acid (Gluma Etch, Heraeus, Hanau, Germany) for 60 s. Then, the denture was cemented using the light-cured luting resin (RelyX Veneer, 3M ESPE, St. Paul, USA). The excessive cement was removed and the margins of the denture were polished. Before going home, the patient was instructed how to brush the denture and maintain oral health.

### 2.3. Correction of the Pontic

**Figure 7:**
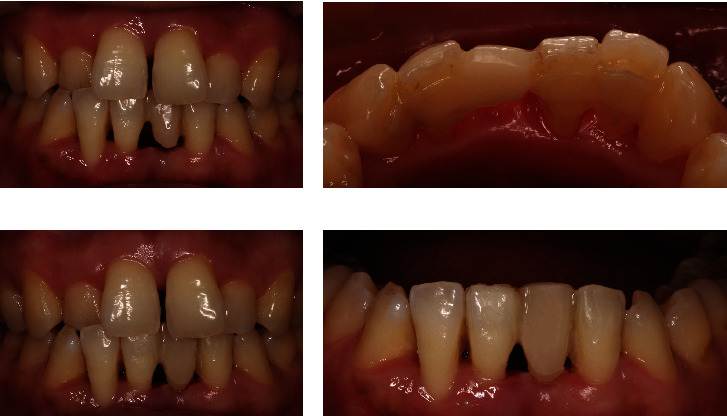
The gingival side of the pontic was extended orally with light-cured resin when the extraction socket was healed. (a) Labial view of the denture before the correction of the pontic. (b) Lingual view of the denture before the correction of the pontic. (c) Labial view of the denture after the correction of the pontic. (d) Improvement in the shape of the pontic and its contact with the mucosa.

Three months later, the patient returned without any complaints about the denture. The extraction socket was healed. And the space under the gingival bottom of the pontic became larger. In order to improve the aesthetics, the gingival side of the pontic was extended with light-cured resin and formed a modified ridge lap pontic while the residual alveolar ridge was covered with a layer of polytetrafluoroethylene (PTFE), and the lip and tongue were isolated by cotton rolls (Figure 7).

### 2.4. Follow-Up and Maintenance

**Figure 8:**
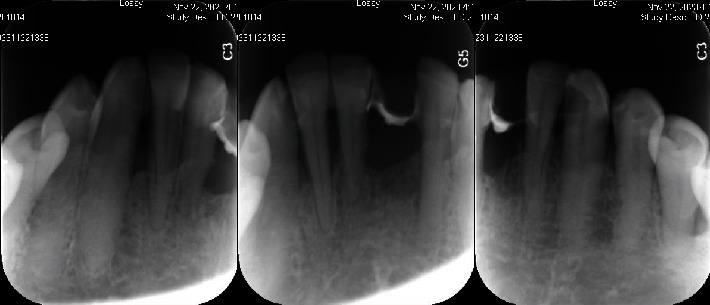
Periapical film follow-up after one-and-a-half years of restoration.

The patient underwent regular follow-up maintenance and periodontal treatment after restoration. On the examination day of the one-and-a-half-year postrestoration, it was found that the restoration was intact without any defects, with tight edges and good occlusal contact. The periapical film (Figure 8) showed that bone resorption in the mesial and distal regions of the three incisors stopped, and there was a slight increase in bone height. The bone height in the mesial and distal regions of the two canines remained stable and did not decrease.

## 3. Discussion

Faced with the requirement of periodontal splint for the loose remaining mandibular anterior teeth, common methods currently include adhesive fixation method and light-cured resin splints. However, for the missing mandibular central incisor, there was a lack of restorations with satisfactory results. Usually, the only option was a removable partial denture restoration. Subsequently, the patient would have to tolerate the foreign body sensation of the denture base or major connector, the inconvenience of removing and inserting the denture, and the aesthetic impact of placing metal clasps and thus exposing the metal.

This digitally fabricated FPD allowed the patient to not only have periodontal splints to hold loose teeth in place, but the missing tooth was also restored with a fixed denture. In addition, this restoration method made it possible to provide immediate rehabilitation to the patient at the same visit of the tooth extraction. Compared to the traditional impression, the intraoral digital scans brought many benefits for the fitting of FPD without any unfavorable force imposed on the loose teeth. This CAD-CAM technique will also make the reproduction of the prosthesis much easy in case the fracture happens after the cementation and the remaking can even start as soon as the patient phones to report the damage. A new denture will be well prepared before the patient's return visit.

Based on the results of a 4-year clinical study that RBFPDs obtained relatively satisfactory survival rate and improved periodontal condition when used as periodontal splints and fixed prostheses to replace missing mandibular anterior teeth, RBFPD would be a reasonable alternative for patients with one or two absent mandibular teeth and periodontally compromised mobile adjacent teeth [12]. In addition, there are some advantages compared to all-ceramic RBFPDs, such as the economic cost, the bonding convenience, and the possibility of direct repairment in the mouth [6–9, 12]. However, whatever fabrication technique, light-cured or milling, the main disadvantages of composite-based RBFPDs are their relatively low strength and esthetic appearance to be improved. Additionally, other variables can have a significant influence on long-term durability of dental frameworks, such as depth of cure [14], curing type [15], and interface contamination [16]. In addition, milling tools and cutting stress may also have some effects on surface quality and stiffness of CAD-CAM resin materials [17]. Therefore, future studies are needed involving also these important factors. And further reports are needed in order to increase the long-term knowledge about CAD-CAM materials.

## 4. Conclusions

The CAD-CAM RBFPDs provided the possibility not only to replace the lost teeth immediately after extraction but also to splint the adjacent mobile simultaneously.

### 4.1. Limitations

The effectiveness of this minimally invasive rehabilitation method needs to be verified by long-term observation, including the preservation time of the RBFPD, the fixation effect on loose teeth, and the impact on oral hygiene maintenance.

## Figures and Tables

**Table 1:** The process of FPD restoration treatment.

**Times of visits**	**The work in the clinic**	**The work in the laboratory**
1	1. Planning the treatment 2. Intraoral digital scan impression	1. Digital design of FPD 2. Fabrication of FPD
2	1. Extraction of the mandibular left central incisor 2. Immediate cementation of FPD after the extraction	
3	Correction of the pontic of the FPD	
4	Follow-up every 3 months	

## Data Availability

This study was registered in the China Medical Research Registration System (http://www.medicalresearch.org.cn). The data is also available on request.
